# Mechanisms of Cyclic Nucleotide Phosphodiesterases in Modulating T Cell Responses in Murine Graft-versus-Host Disease

**DOI:** 10.1371/journal.pone.0058110

**Published:** 2013-03-06

**Authors:** Michael Weber, Corinna Lupp, Pamela Stein, Andreas Kreft, Tobias Bopp, Thomas C. Wehler, Edgar Schmitt, Hansjörg Schild, Markus P. Radsak

**Affiliations:** 1 Institute for Immunology, Johannes Gutenberg-University Medical Center, Mainz, Germany; 2 Institute of Pathology, Johannes Gutenberg-University Medical Center, Mainz, Germany; 3 Department Internal of Medicine III, University Medical Center, Johannes Gutenberg-University, Mainz, Germany; Leiden University Medical Center, The Netherlands

## Abstract

Graft-versus-host disease (GvHD) is a key contributor to the morbidity and mortality after allogeneic hematopoetic stem cell transplantation (HSCT). Regulatory Foxp3^+^ CD4^+^ T cells (T_reg_) suppress conventional T cell activation and can control GvHD. In our previous work, we demonstrate that a basic mechanism of T_reg_ mediated suppression occurs by the transfer of cyclic adenosine monophosphate (cAMP) to responder cells. Whether this mechanism is relevant for T_reg_ mediated suppression of GvHD is currently unknown. To address this question, bone marrow and T cells from C57BL/6 mice were transferred into lethally irradiated BALB/c recipients, and the course of GvHD and survival were monitored. Transplanted recipients developed severe GvHD that was strongly ameliorated by the transfer of donor T_reg_ cells. Towards the underlying mechanisms, *in vitro* studies revealed that T_reg_ communicated with DCs via gap junctions, resulting in functional inactivation of DC by a metabolic pathway involving cAMP that is modulated by the phosphodiesterase (PDE) 4 inhibitor rolipram. PDE2 or PDE3 inhibitors as well as rolipram suppressed allogeneic T cell activation, indirectly by enhancing T_reg_ mediated suppression of DC activation and directly by inhibiting responder T cell proliferation. In line with this, we observed a cooperative suppression of GvHD upon T_reg_ transfer and additional rolipram treatment. In conclusion, we propose that an important pathway of T_reg_ mediated control of GvHD is based on a cAMP dependent mechanism. These data provide the basis for future concepts to manipulate allogeneic T cell responses to prevent GvHD.

## Introduction

For patients with high risk hematological malignancies allogeneic hematopoetic stem cell transplantation (HSCT) is the only curative treatment option [1]. The therapeutic principle of HSCT relies on a graft versus leukemia (GvL) or graft versus tumor (GvT) effect generated by donor lymphocytes that specifically recognize and eliminate malignant cells in the recipient [2]. However, after HSCT additional immune responses may occur against healthy tissues creating graft-versus-host disease (GvHD), an important contributor to transplant related morbidity and mortality [3]. To improve the feasibility of HSCT, it will be crucial to gain the ability to guide immune responses in the desired way maintaining anti-viral and anti-tumor responses while controlling undesired responses, namely GvHD.

Naturally occuring regulatory T cells (T_reg_) are responsible for maintaining peripheral self tolerance [4], but they may also play a role in the failure to control tumor growth as T_reg_ cell depletion can facilitate tumor rejection [5], [6]. In the context of HSCT, T_reg_ cells have been shown to control GvHD [7]–[9], while on the other hand preserving GvT reactions [10]. However, the strategies to control effector T or T_reg_ cell activity might be relevant since stringent inhibition of both may result in poor tumor outcome [9]. In addition, current clinical protocols demonstrate the feasibility and safety of T_reg_ cell transfer in humans [11], possibly opening T_reg_ based treatment options for patients beyond experimental settings in the near future. Hence, it is important to understand the relevant mechanisms of T_reg_ mediated suppression in HSCT.

Donor T cells are activated by conventional CD11c^+^ dendritic cells (DC) in the HSCT recipient [12]–[14]. In this context, the blockade of costimulatory molecules induces transplantation tolerance that is mediated by T cell anergy or T_reg_ subpopulations depending on the particular model used [15]–[17]. In general, T_reg_ mediated suppression occurs by cytokine independent [18]–[20], but contact dependent ways, i. e. via the glucocorticoid induced tumor necrosis factor receptor (GITR) [20], CTLA-4 or membrane-bound TGF-β [21]. Beyond this, the transfer of cyclic adenosine monophosphate (cAMP) to target cells via gap junction intercellular communication (GJIC) is a key mechanism of T_reg_ mediated suppression [22], [23], also important in T_reg_-DC interaction that occurs by a direct contact (cAMP) dependent and by a contact independent pathway [24].

To examine the suppressive mechanisms utilized by T_reg_ cells in the context of HSCT, we investigated a MHC mismatched mouse model of acute GvHD. We confirm an important role for T_reg_ cells in ameliorating GvHD and show that T_reg_ cells communicate with DC via a GJIC and a cAMP dependent mechanism, resulting in the cooperative suppression of allogeneic MLR using the cAMP elevating drugs/phosphodiesterase (PDE) inhibitors. Conversely, we observed cooperative amelioration of GvHD by T_reg_ transfer and treatment with the PDE inhibitor rolipram. These results suggest that multimodal strategies combining T_reg_ cellular transfer with cAMP modulating drug therapies may be a new treatment strategy for acute GvHD. This may contribute to future concepts in improving the feasibility and efficacy of HSCT.

## Materials and Methods

### Reagents

Biotinylated anti-CD25 (7D4) was purchased from BD (Heidelberg, Germany), PE- conjugated streptavidin was obtained from Dianova (Hamburg, Germany), anti-PE beads were obtained from Miltenyi Biotec (Bergisch Gladbach, Germany). The following additional mAbs were used: anti-CD3 mAb (145-2C11) and anti-CD28 mAb (37.51). If required, mAbs were affinity purified using protein G–Sepharose (GE Healthcare, Munich, Germany). Mouse recombinant IL-2 was affinity purified. Rolipram was purchased from Sigma-Aldrich (Taufkirchen, Germany), Cilostazol was obtained from Tocris Biosciences (Bristol, UK), BAY-60-7550 was purchased from Santa Cruz Biotechnology (Santa Cruz, USA).

### Mice

C57BL/6, BALB/c, and B6.SJL (CD45.1^+^) mice were obtained from Charles River Laboratories and bred in a specific pathogen-free colony in the animal facility of the JGU Mainz. All animal procedures were performed in accordance with the institutional guidelines and approved by the responsible national authority (National Investigation Office Rheinland-Pfalz, Appoval ID: AZ 23 177-07/G07-1-009).

### Cell Purification and Culture

Splenic DC from BALB/c mice were purified by density centrifugation as described previously [25] or bone marrow derived DC (BMDC) generated from bone marrow as described previously [26] and used as stimulators for allogeneic mixed lymphocyte reactions (MLR). Briefly, bone marrow from 6–8 weeks old mice was cultured in Iscovés medium supplemented with 5% fetal calf serum (FCS, inactivated at 56°C), 1% glutamine, 1% sodium pyruvate and GM-CSF (50 ng/ml). These cells were typically >85% CD11c^+^ as determined by flow cytometry. Furthermore, CD11c^+^ cells were CD80^low^. Medium was changed on days 2 and 4. Cells were used for the experiments at day 6 as immature DC.

Responder T cells from C57BL/6 splenocytes were purified by anti-CD90.2 conjugated magnetic beads (Miltenyi Biotec, Bergisch-Gladbach, Germany) according to the manufacturer’s instructions. T_reg_ depletion of CD90.2^+^ T cells was performed with biotinylated anti-CD25 (7D4) and magnetic streptavidin-conjugated microbeads (Miltenyi Biotec).

T_reg_ were isolated as described previously [22] using biotin-conjugated anti-CD25 mAb (7D4), PE-conjugated streptavidin and magnetic anti-PE microbeads (Miltenyi). CD25 sort was performed twice. CD25^+^-enriched T_reg_ cells were additionally depleted from CD8^+^ T cells, B cells and macrophages using anti-CD8, anti-B220 and anti-MAC1 Dynabeads (Dynal Biotech, Hamburg, Germany). The purity of the resulting CD25^+^ Foxp3^+^ T_reg_ cells was typically >95%. In some experiments, T_reg_ cells were preactivated by a combination of plate-bound anti-CD3 mAb (145-2C11; 3 µg/ml) and anti-CD28 mAb (37.51; 3 µg/ml) in presence of 1000 U/ml IL-2 (Proleukine from Novartis, Nuremberg, Germany). Cells were splitted on day 3 without further anti-CD3/anti-CD28 stimulation and harvested on day 5 and used as preactivated T_reg_ (pre T_reg_). In some experiments, T_reg_ cells were labelled with calcein (1 µM, from Molecular Probes, Eugene, Oregon, USA) for 30 min. at 37°C, and washed twice in medium before use. Where indicated the GJIC inhibitor GAP27 (300 µM, from Biozol, Eching, Germany) was added [22].

For MLR, 2×10^5^ CFDA-SE labelled (1 µM, from Molecular Probes, Eugene, Oregon, USA) responder T cells per well were added to DC and/or T_reg_ in titrated ratios as indicated and cultured for 4 days in 96 well plates before cell harvest. Cells were washed, labelled with specific mAbs and analyzed for CFDA-SE dilution as indicator for proliferation by flow cytometry. Alternatively, where indicated activation of responder T cells in MLR was assessed by ^3^H-thymidine incorporation as follows: unlabeled C57BL/6 T cells (CD90.2^+^ T cells purifed by magnetic sorting) were added to the MLR culture. ^3^H-thymidine (0.5 µCi/well) was added on day 3 of the culture, cells were harvested 18 h later for assement of ^3^H-thymidine uptake by β scintillation counting.

Where indicated, DC or T_reg_ were fixed after 4 h of coincubation as follows: Cells were washed twice in PBS and suspended in 0.1% glutaraldehyde (Sigma-Aldrich, St. Louis, USA) in PBS. After 30 seconds lysine (0.2 M) and FCS were added to quench the reaction. The cells were washed twice in medium and added to secondary cultures as indicated.

### Flow Cytometric Analyses and Calcein Transfer

After coculture cells were harvested, washed and stained for 30 minutes with antibodies. The following mAbs were used for analyses by flow cytometry: CD8 (clone 53-6.7), CD11c (N418), CD80 (16-10A1), CD86 (GL1), CD40 (3/23), B7-H1 (MIH5), B7-DC (122), MHCII (M5/114.15.2), CD90.2 (53-2.1), CD45.1 (clone A20; all from eBiosciences or BD Pharmingen, Hamburg, Germany). Viability was determined by propidium iodide.

For calcein transfer assays, calcein labelled pre T_reg_ and BMDC were cocultured in a ratio of 5∶1 for 4 hours and subsequently harvested for FACS analysis.

Intracellular cAMP levels were assessed by a cAMP specific mAb (SMP486, Abcam, Cambridge, UK) [27] labelled with fluorescein (Lightning-Link fluorescein conjugation kit, Innova Biosciences, Cambridge, UK). After 4 h of coculture of preT_reg_ and BMDC, the cell surface was stained with anti-CD11c and anti-CD90.2, cells were fixed with 4% PFA and permeabilized with saponine and subsequently stained with fluorescein-labled anti-cAMP or an fluorescein-labled isotype control.

All analyses were performed with a FACSCanto or a LSRII flow cytometer and FACSDiva software (BD) or FlowJo (Tree Star Inc.).

### Purification of DC after Cocultures

After 4 h of coculture of preT_reg_ and DC, cells were separated by anti-CD11c microbeads (Miltenyi Biotec) according to the manufacturers instructions. Purity of CD11c^+^ MHCII^+^ double positive cells was assessed by flow cytometry and was >97%.

### Cyclic AMP ELISA

To assess cytosolic cAMP concentrations, DC were washed three times in PBS, lysed in 0.1 N HCl (1×10^7^/ml) and a cAMP specific ELISA was performed (Direct cAMP EIA kit, Enzo Life Sciences, Lörrach, Germany). The resulting cAMP concentration (pmol/ml) was calculated for 1×10^6^ cells.

### IL-12 ELISA

Splenic DC were stimulated in triplicate wells (96-well plate) for 2 days as indicated. The cell free supernatants were collected and frozen at −20°C until required. The concentration of IL-12p40/p70 was assessed in the supernatants by sandwich ELISA (obtained from BD Pharmingen), according to the manufacturer’s instructions.

### Bone Marrow Transplantations

Mice were transplanted following a standard protocol. At day −1 recipient animals received total body irradiation (TBI, 850 cGy for BALB/c) from ^137^Cs source (OB58-BA, Buchler, Braunschweig, Germany). On day 0, allogeneic donor T cell depleted bone marrow cells (TCD-BM; 5×10^6^ cells per animal) and 5×10^5^ CD90.2^+^ T cells (BM/T) were transferred by intravenous injection. Where indicated T_reg_ cells (5×10^5^ cells per animal) were co-injected. The animals were maintained under specific pathogen free conditions and received antibiotics (Sulfadoxin-Trimethoprim 1 g/ml in the drinking water) post transplantation.

Bone marrow cells were isolated from femura and tibiae, erythrocytes were lysed, T cells were depleted by using magnetic anti-CD90.2 microbeads (Miltenyi Biotec). Cells were washed twice and injected suspended in PBS.

### Assessment of GvHD

The degree of systemic GvHD was examined at least every other day using a scoring system as described elsewhere [28]. The scoring system includes 5 clinical parameters: weight loss, posture, activity, fur texture, and skin integrity. The scoring system was modified as pointed out in Table 1 using four instead three categories for weight loss to reduce animal suffering. Animals with a score of 2 or greater were inspected daily. To further limit animal suffering, mice with severe symptoms of GvHD, such as severe weight loss, reduced activity, hunched posture and scrubby fur texture as determined by clinical scores equal or greater than 6, were immediately euthanized by CO_2,_ as required by the institutional animal ethics guide lines and the day subsequent to death determined as the following day.

**Table 1:** GvHD clinical Score.

Score	Weight loss	Skin	Activity	Posture	Fur texture
0	<5%	normal	normal	normal	normal
1	5–10%	flaked	reduced	hunched	scrubby
2	10–20%	explicit fur loss	inactive	severely hunched	severely scrubby
3	>20%				

Mice were evaluated for clinical signs of GvHD twice weekly according to Table 1 for each category. Individual scoring points were cumulated. Mice were sacrificed when exceeding a cumulative score ≥6.

### Histology

Mice were sacrificed on day 10 after transplantation, fur on the back was removed mechanically and biopsies of the skin were taken, fixed in 4% buffered formalin, parafine wax embedded, sectioned and stained with haematoxylin and eosin according to standard protocols.

### Statistical Analysis

Statistical analyses comparing two groups of assumed Gaussian distribution were performed by a two-tailed Student’s *t-*test using GraphPad Prism (version 5.0a for Mac OSX, GraphPad Software, San Diego California USA, www.graphpad.com). Alternatively, a Mann-Whitney *U-*test was used as indicated for other distributions. For differences in survival, the indicated groups were compared and analyzed by Mantel-Cox test. For all analyses, p<0.05 was considered significant.

## Results

### Regulatory T cells Suppress Graft-versus-Host Disease

**Figure 1:**
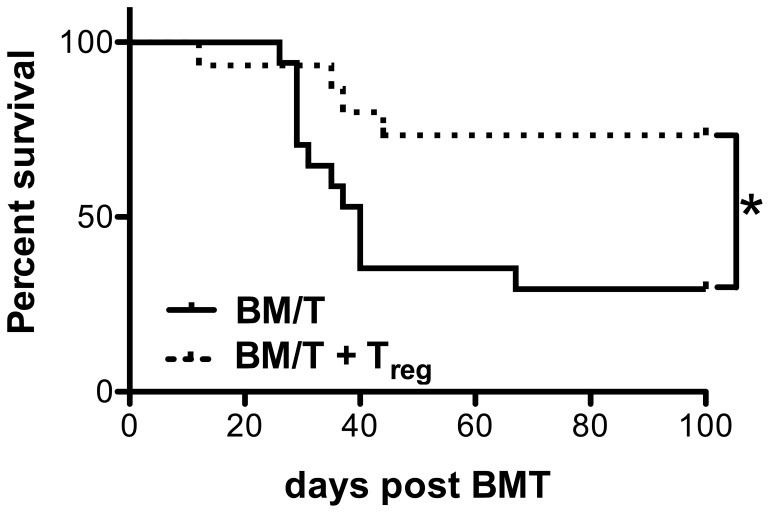
T_reg_ cells suppress GvHD. BALB/c recipient mice were lethally irradiated (8,5 Gy) and received allogeneic C57BL/6 TCD-BM (5×10^6^ cells) and Thy1.2^+^ T cells (5×10^5^ cells, WT, black line, n = 17). A second group additionally received C57BL/6 T_reg_ cells (5×10^5^ cells, WT+WT T_reg_, broken line, n = 15). Survival was monitored for 100 days. Combined data from 3 independent experiments are shown. (*) indicates for *P*<0,05 according to a Mantel-Cox-Test.

In HSCT, it has been clearly demonstrated that T_reg_ cells can control GvHD [3], [7]. However, it is not clear how this is mediated in detail and multiple mechanisms of T_reg_ mediated suppression have been described. To address this issue, we used a MHC mismatched HSCT model where BALB/c wild type hosts are lethally irradiated and transplanted with bone marrow and purified T cells from C57BL/6 mice. As depicted in Fig. 1, the animals succumbed to GvHD with a median survival of 40 days. Upon transfer of T_reg_ cells, we observed protection from lethal GvHD in the transplanted animals (median survival not reached, p<0.001) which is consistent with previous reports [7].

### Regulatory T cells Communicate with Allogeneic Dendritic Cells via Gap Junctions and Mediate a Suppressive Dendritic Cell Phenotype

**Figure 2:**
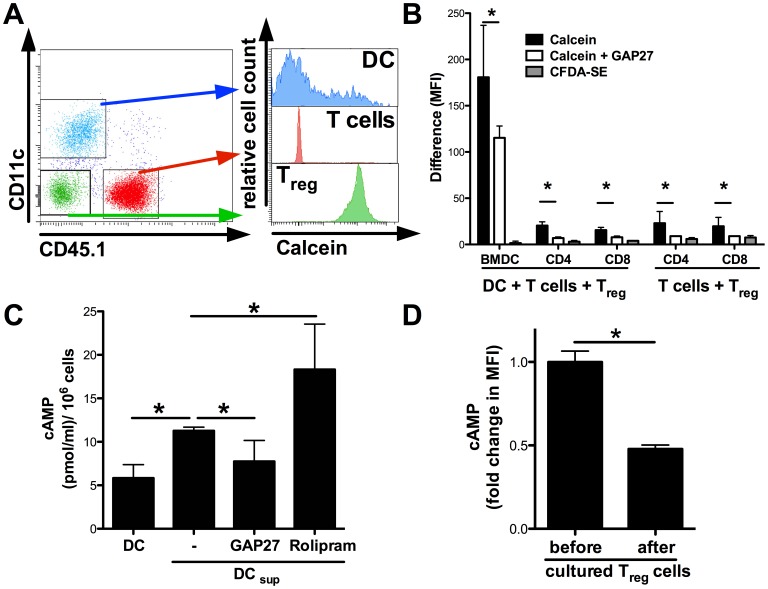
T_reg_ cells communicate with allogeneic DC directly via cell-to-cell contact and GJIC dependent manner. Bone marrow derived dendritic cells (BMDC from BALB/c mice, 3×10^4^ per well) were cocultured with B6.SJL CD45.1^+^ T cells and C57BL/6 CD45.1^−^ calcein-labelled pre T_reg_ cells for 20 h in a 1∶1∶3 ratio. (A) Transfer of green fluorescent calcein after 20 h was analyzed by gating on CD45.1^−^CD11c^+^ DC, CD45.1^−^CD11c^−^ T_reg_ cells and CD45.1^+^CD11c^−^ T cells, respectively. (B) Differences of median fluorescence intensity (MFI) among BMDC, CD4^+^ and CD8^+^ T cells was assessed. Where indicated T_reg_ cells were preincubated with the gap junction inhibitor GAP27 (300 ng/ml). CFDA-SE (1 µM) labelled T_reg_ cells were used as a control. (C) BALB/c BMDC were left untreated or cocultured with C57BL/6 pre T_reg_ cells in an 1∶1 ratio in presence of soluble anti-CD3-mAb (3 µg/ml). Where indicated GAP27 (300 ng/ml) or rolipram (300 nM) were added. After 4 h suppressed DC (DC _sup_) were purified by CD11c specific MACS. Intracellular cAMP concentration was assessed by a specific ELISA after lysis of the cells. (D) Changes in cAMP levels after a 4 h coculture with BALB/c BMDC were mesured by flow cytometry in C57BL/6 preT_reg_. (*) indicates significant differences by Mann-Whitney *U-*test. The data shown are representative for 2 independent experiments in duplicate or triplicate wells.

In our previous work, we have characterized the principle ability of T_reg_ cells to inhibit T cell responses by suppression of DC activation. In continuation of this work, we were interested whether these mechanisms of T_reg_ mediated inhibition of DC activation are also relevant for the suppression of allogeneic T cell activation and the initiation of GvHD. We and others have demonstrated before that T_reg_ cells utilize a GJIC dependent pathway to suppress CD8^+^ T cell, CD4^+^ T cell and DC activation [22], [24], [29]. Therefore we asked for the relevant T_reg_ interaction partners in the setting of alloreactive T cell activation. We used the fluorescent dye calcein that can be transferred from one cell to another only via GJIC [24], [30]. T_reg_ cells were labelled with calcein and cocultured with DC and responder T cells for 4 hours. Subsequently, the amount of green fluorescent calcein was quantified in the lymphocyte or DC gate after exclusion of conjugates and doublets as illustrated in Fig. 2A. In the quantitative analysis (Fig. 2B), we detected a transfer of calcein from T_reg_ to T cells. However, the relative amount transferred to DC was significantly higher. As a control, we co-incubated the cells with T_reg_ cells that had been labelled with CFDA-SE, a green fluorescent dye that cannot be passed over from one cell to another by GJIC [29]. In this situation, we did not detect any transfer of fluorescence to DC or T cell populations (Fig. 2B, grey bars). The transfer of calcein was inhibited by the GJIC inhibitory peptide GAP27 (Fig. 2B, open bars) suggesting that T_reg_ cells and DC truly communicate via GJIC also in the context of allogeneic T cell activation.

To directly address whether this contact also affects the levels of intracellular cAMP in DC, we coincubated DC with T_reg_ cells for 4 h and separated the cells afterwards by magnetic sorting. Subsequently, we used a cAMP specific ELISA to quantify the amounts of cAMP in DC [22], [24]. As depicted in Fig. 2C, DC harbour low levels of intracellular cAMP at baseline. Consistent with our previous results in the syngenic setting [24], cAMP levels in DC were significantly increased upon contact with T_reg_ cells. Importantly, the increase in cAMP after contact with T_reg_ cells was partly inhibited in the presence of the GJIC inhibitor GAP27 further supporting the relevance of a GIJC dependent pathway in this situation. Moreover, intracellular cAMP was even further increased in the presence of the PDE4 inhibitor rolipram. In addition, we analyzed the intracellular cAMP content in T_reg_ cells before or after coincubation with DC by flow cytometry [27] and found that the cAMP levels in T_reg_ cells were significantly decreased after coculture, further supporting the idea of cAMP transfer to DC (Fig. 2D).

**Figure 3:**
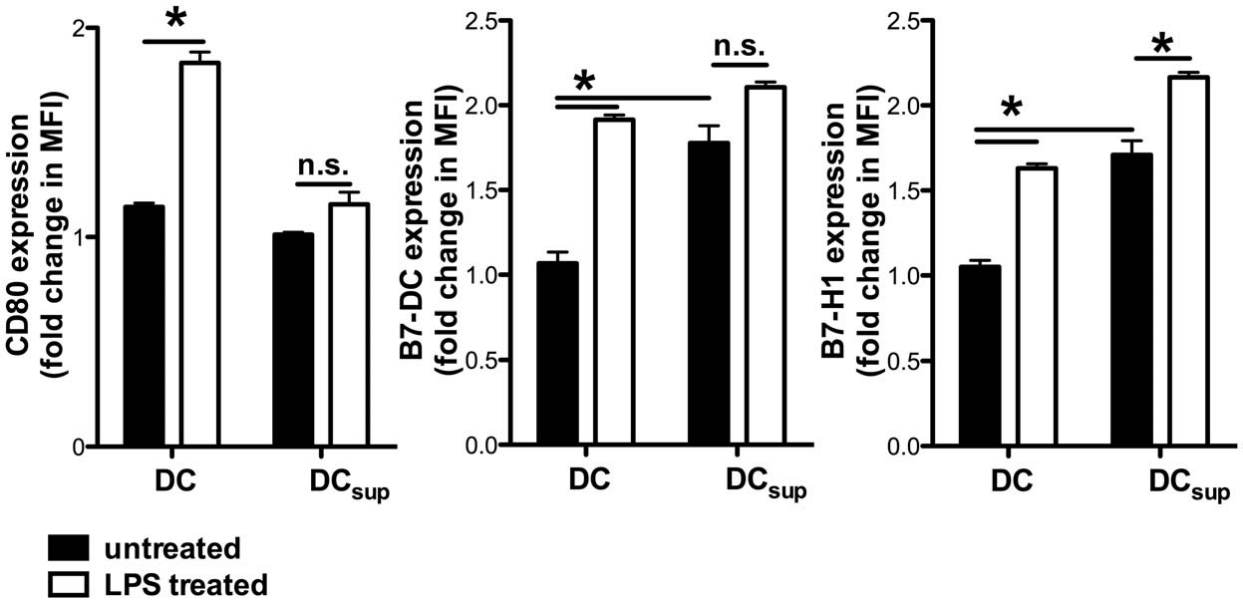
T_reg_ cells induce a suppressive DC phenotype. BALB/c BMDC were left untreated or stimulated with LPS (100 ng/ml) and cocultured with C57BL/6 T_reg_ cells in an 1∶1 ratio (DC _sup_). For optimal T_reg_ stimulation, a soluble anti-CD3-mAb (3 µg/ml) was added. After 4 h expression of CD80, B7-H1 and B7-DC was determined by flow cytometry gating on CD11c^+^ MHCII^+^ cells. All depicted results were assayed in triplicate wells and are representative of three independent experiments. (*) indicates significant differences by Mann-Whitney test; n.s. – no significant differences.

As a functional consequence of the contact with T_reg_ cells, DC downregulate the costimulatory molecule CD80 while upregulating the inhibitory molecules B7-H1 and B7-DC (Fig. 3 and Fig. S1), as also demonstrated by us previously in a syngenic setting [24]. Taken together, our results suggest that T_reg_ cells induce a suppressive DC phenotype in a cell contact dependent manner that involves cAMP.

### Inhibition of Phosphodiesterases and Regulatory T cells Cooperatively Suppress Allogeneic Mixed Lymphocyte Reactions

We have previously shown that the transfer of cAMP via GJIC from T_reg_ to recipient cells is important for T_reg_ mediated suppression. To enhance this metabolic pathway, the intracellular break down of cAMP may be blocked by inhibitors of PDEs, such as the PDE4 inhibitor rolipram which leads to an enhanced efficacy of T_reg_ mediated suppression *in vitro* and also *in vivo*, as recently demonstrated in an asthma model [23]. Beyond PDE4, also other PDEs can affect T_reg_ functionality as recently demonstrated by Feng and coworkers who find a pivotal role for the inhibition of PDE3 in allograft rejection [31].

**Figure 4:**
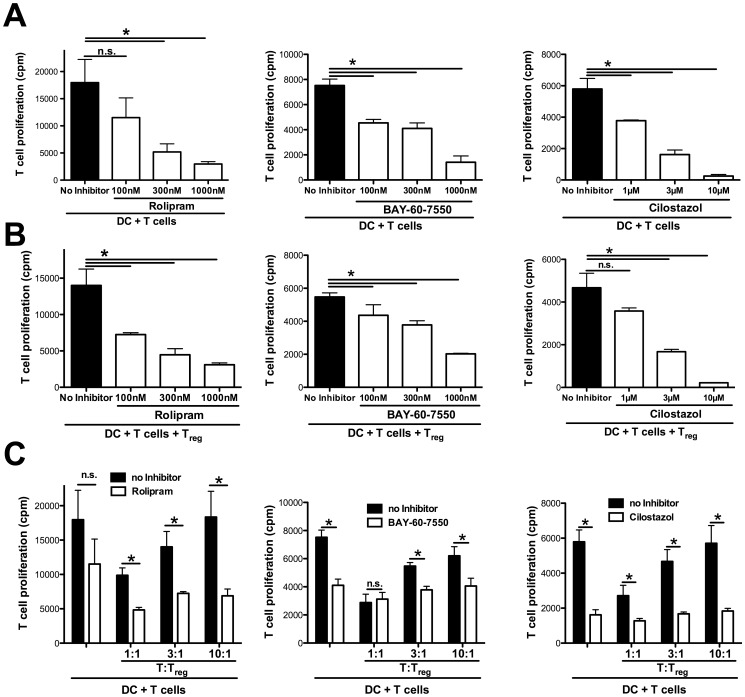
Rolipram enhances the suppressive capacities of T_reg_ cells *in vitro.* (A, B) BALB/c splenic DC (3×10^4^ per well) were cocultured with allogeneic C57BL/6 Thy1.2^+^ T cells (1×10^5^ per well) with titrated amounts of the PDE4 inhibitor rolipram (100 to 1000 nM), PDE2 inhibitor BAY-60-7550 (100 to 1000 nM) or PDE3 inhibitor Cilostazol (1 µM to 10 µM) for 3 days. Cell proliferation was determined after 3 days by ^3^H-thymidine incorporation. (B) DC (3×10^4^ per well) were cocultured with allogeneic C57BL/6 Thy1.2^+^ T cells (1×10^5^ per well) as in (A), but C57BL/6 T_reg_ cells (3×10^4^ per well) were added. (C) DC (3×10^4^ per well) were cocultured with allogeneic C57BL/6 Thy1.2^+^ T cells (1×10^5^ per well) and C57BL/6 T_reg_ cells in indicated ratios for 3 days. Where indicated PDE4 inhibitor rolipram (100 nM), PDE2 inhibitor BAY-60-7550 (300 nM) or PDE3 inhibitor (3 µM) was added and proliferation was assessed after 3 days by ^3^H-thymidine incorporation. All depicted results were assayed in triplicate wells and are representative of three independent experiments. (*) indicates significant differences by Mann-Whitney *U-*test; n.s. – no significant differences.

Therefore we evaluated the role of inhibitors for PDE2 (BAY-60-7550), PDE3 (Cilostazol) or PDE4 (rolipram) in the suppression of allogeneic T cell responses in the presence or absence of T_reg_ cells. As shown in Fig. 4A, the additional presence of each of these inhibitors suppressed MLR induced T cell proliferation in a concentration dependent manner, even when not adding T_reg_ cells. Nevertheless, when T_reg_ cells were added to the MLR, T cell proliferation was further suppressed, either titrating the respective inhibitors (Fig. 4B) or the number of T_reg_ cells in the culture (Fig. 4C).

Since we found T_reg_ dependent as well as independent suppression of MLR by all tested PDE inhibitors, these results suggest that cAMP elevating drugs and T_reg_ cells have cooperative effects despite the fact that PDE isoforms differentially expressed, i. e. PDE3b being highly repressed by T_reg_ cells [32]. This indicates that these drugs mediate suppression of MLR by multiple mechanisms.

### Rolipram Enhances T_reg_ Function and Suppresses DC and Responder T cell Activation

Because we observed similar effects by all PDE inhibitors, we decided to focus our further studies for the underlying mechanisms on the PDE4 inhibitor rolipram where we have observed cooperative effects with T_reg_ cells in an asthma model [23]. We asked whether rolipram directly suppresses the activation of responder T cells or indirectly, i. e. by modulating the T_reg_/DC interaction. Firstly, we incubated DC in the presence or absence of rolipram and found that IL-12 production was diminished in escalating doses of rolipram, while we did not observe a significant impact on the activation (CD80, CD86 or CD40) phenotype or viability (Fig. S2). Next, we cocultured DC with allogeneic T_reg_ cells in the additional absence or presence of rolipram and fixed the cells after 4 h with glutaraldehyde, as illustrated in Fig. S3A. Subsequently, the cells were cultured with allogeneic responder T cells, again in the absence or presence of rolipram. As shown in Fig. S3B, fixed DC induced the activation of allogeneic T cells, although markedly less than viable DC depicted in comparison. Nevertheless, preactivation of DC with the TLR4 agonist LPS resulted in a significant increase in T cell activation that was suppressed in the presence of T_reg_ cells (Fig. S3C). It is important to notice that the 4 h coincubation period of viable DC with T_reg_ before fixation was necessary and sufficient for the impairment of subsequent allogeneic T cell activation since coincubation of DC with fixed T_reg_ cells had no impact on the MLR (Fig. S3D).

**Figure 5:**
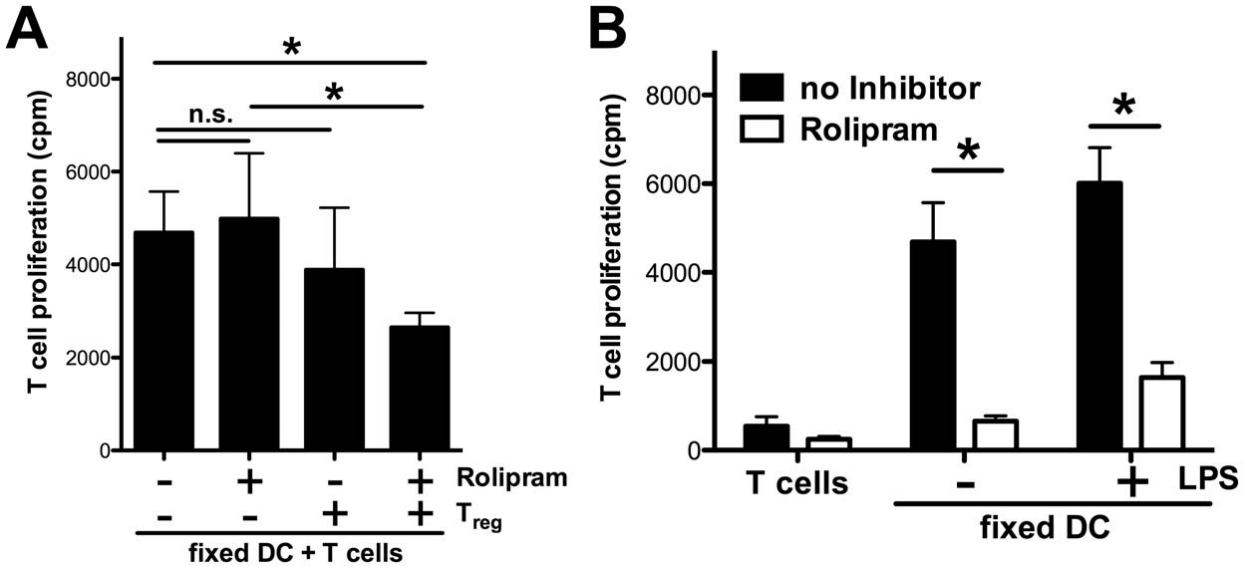
Rolipram suppresses MLR directly by inhibiting T cell prolifation and indirectly by modulating DC/T_reg_ interaction. (A) BALB/c BMDC were cultured in absence or presence of C57BL/6 T_reg_ cells (1∶1 ratio) and rolipram (300 nM) for 4 h. Anti-CD3 (3 µg/ml) was added to every well. After fixation cells were cocultured with C57BL/6 Thy1.2^+^ T cells in a 10∶1 ratio for 3 days. Proliferation was determined by ^3^H-thymidine incorporation. (B) BALB/c BMDC were stimulated with LPS (100 ng/ml) for 4 h or left untreated and subsequently fixed with glutaraldehyde. C57BL/6 Thy1.2^+^ CD25^−^ T cells were cocultured with LPS stimulated or unstimulated fixed BMDC (1×10^4^ per well) for 3 days in a 10∶1 ratio. All depicted results were assayed in triplicate wells and are representative of three independent experiments. (*) indicates significant differences by Mann-Whitney *U-*test; n.s. – no significant differences.

To further support a role for cAMP in this situation, we added the PDE4 inhibitor rolipram into the MLR culture. Under these stringent conditions, we did not observe any direct suppressive effect of rolipram on DC in terms of inhibition of T cell activation (Fig. 5A). Upon contact of DC with T_reg_ alone, this brief incubation period also did not result in significant suppression of T cell activation. In contrast, only in the presence of rolipram and T_reg_ cells the capability of DC to induce allogeneic T cell activation was significantly suppressed.

To clarify whether rolipram also directly affects the activation of responder T cells, we coincubated fixed DC with allogeneic responder T cells in the additional presence or absence of rolipram demonstrating that rolipram also directly inhibited allogeneic T cell proliferation (Fig. 5B). These results indicate that rolipram suppresses MLR by at least three distinct mechanisms: the inhibition of IL-12 production by DC, the direct inhibition of allogeneic T cell proliferation and a T_reg_/DC dependent pathway involving cAMP that mediates a diminished alloreactive capacity of DC.

### Rolipram and Regulatory T cells Cooperatively Ameliorate Acute Graft-versus-Host Diseases

**Figure 6:**
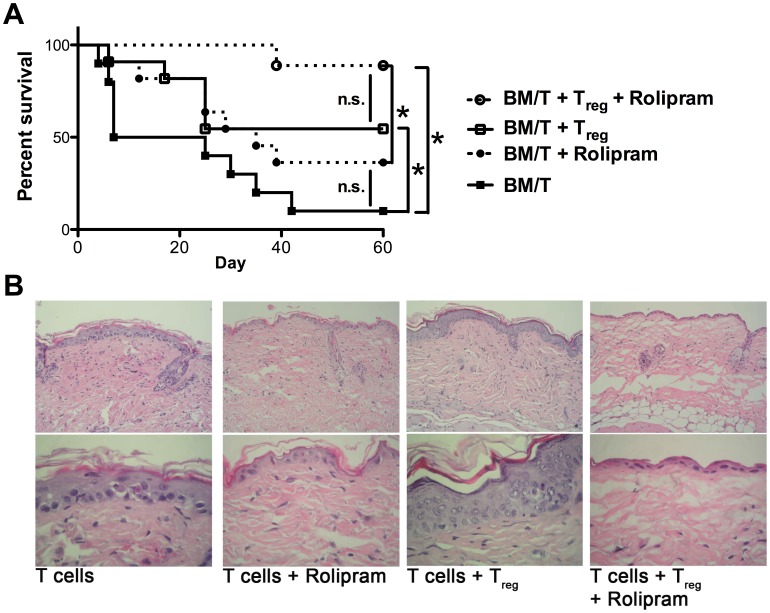
Rolipram enhances suppressive capacities of T_reg_ cells *in vivo and ameliorates GvHD*. (A) BALB/c mice were lethally irradiated (8,5 Gy) and received either TCD bone marrow (5×10^6^ cells) and Thy1.2^+^ CD25^−^ T cells (5×10^5^ cells) from C57BL/6 donors (n = 10, filled squares), Thy1.2^+^ CD25^−^ T cells plus T_reg_ cells (1∶1 ratio, n = 10, open squares), Thy1.2^+^ T cells plus rolipram (n = 10, filled circles) or Thy1.2^+^ T cells plus T_reg_ cells and rolipram (n = 9, open circles). Rolipram (0.3 mg/kg) was injected *i.p.* on days 0 to 20 once per day. Results show the combined survival data from 2 independent experiments (* *p*<0.0005 by Mantel-Cox test). (B) Transplantation and rolipram treatment were performed as descibed above, but the mice were sacrificed on day 10. Representative skin sections from the back were stained with haematoxylin and eosin. The upper pictures are showing an overview (original magnification 200×), the lower pictures are showing a detailed view (original magnification 630×).

To see whether these results obtained by *in vitro* experiments are also relevant *in vivo*, we performed allogeneic BMT, similar to the above described experiments, in the absence or presence of T_reg_ cells and additionally treated the mice with the PDE4 inhibitor rolipram. In contrast to the experiments shown in Fig. 1, we used C57BL/6 derived CD90.2^+^ T cells that had been T_reg_ depleted using a CD25 specific antibody (clone 4D7) for transplantation in order to exclude rolipram driven effects on T_reg_ cells present in the CD90.2^+^ T cell graft. As shown in Fig. 6A and Fig. S4, in mice transplanted with T_reg_ depleted T cells alone we observed acute and lethal GvHD (filled squares, median survival 7 days) comparable to the experiments shown in Fig. 1. Upon the additional transfer of T_reg_ cells (open squares, median survival 29 days), the onset of GvHD was delayed, but we observed an increased mortality (compare Fig. 1: median survival not reached), most likely due to a durable protective effect by an increased number of T_reg_ cells in the initial (not CD25 depleted) transplantation setting. Interestingly, the mice treated with the PDE4 inhibitor rolipram alone showed a prolonged median survival (Fig. 6, filled circles, median survival 25 days). However, only the group of mice that received T_reg_ and rolipram was almost completely protected from lethal GvHD (open circles, median survival not reached). Additional histology skin sections as a classical target organ showed typical GvHD findings, i.e. focal spongiosis of the epidermis associated with only scant lymphoid cell infiltration of the dermis and scattered apoptotic keratinocytes, that appeared less prominent in mice that had received T_reg_ and rolipram (Fig. 6B).

Collectively, these results confirm and extend the concept that T_reg_ mediated suppression of T cell responses involves a cAMP dependent pathway, which can be manipulated by drugs like rolipram.

## Discussion

Utilizing donor derived T_reg_ cells to control GvHD has a great potential to decrease the morbidity and mortality after allogeneic HSCT. The basic capability of T_reg_ cells for this has been demonstrated in experimental settings [7], [10], [11] and is confirmed by our results. However, along with current studies that provide evidence for the clinical feasibility of T_reg_ based immunotherapy [11], it becomes increasingly urgent to clarify the relevant mechanisms of T_reg_ mediated suppression in the context of GvHD in order to be able to define the risks, limits and potentials of such treatments.

Multiple T_reg_ subpopulations and mechanisms of T_reg_ mediated suppression have been described [33], [34]. Inducible T_reg_ cells can suppress GvHD in an antigen specific manner, as recently demonstrated [35]. However, in our current work we followed up on previous observations demonstrating that a major mechanism of suppression by naturally occurring T_reg_ cells is mediated by a pathway involving the transfer of cAMP via GJIC. Current concepts of GvHD priming highlight the role of host derived DC populations in the initiation phase of GvHD [3], [12], [13] and the interaction with T_reg_ cells in this context [36]. Concerning the potential mechanisms of T_reg_ mediated suppression in the context of GvHD, we have recently demonstrated a contact and cAMP dependent pathway of T_reg_ mediated suppression of CD4^+^ T cells [22]. This metabolic pathway is also important for the suppression of DC activation as shown by us *in vitro* [24] as well as *in vivo* as demonstrated in a model for contact hypersensitivity [29] and asthma [23]. Therefore we sought to evaluate this cAMP dependent pathway in the context of allogeneic T cell activation. We found that indeed T_reg_ cells preferentially make contact with DC and transfer the small fluorescent dye calcein as surrogate for cAMP to DC rather than directly to effector T cells. In line with this and consistent with our previous results obtained in the syngeneic setting [24], DC downregulate costimulatory molecules coincidently with elevated levels of cAMP that can be blocked in part by the GJIC inhibitor GAP27. The significance of this mechanism in suppression of GvHD is underpinned by Yi et al. demonstrating that the interaction of host APC with T_reg_ cells via CD80/B7H1 is important for T_reg_ expansion post HSCT [37]. To evaluate the functional contribution of cAMP in this situation, we used three different PDE inhibitors, all confirming previous results obtained with rolipram for enhanced cAMP levels and suppressive function of T_reg_ cells *in vitro* and also *in vivo* [22], [23]. Apparently, all PDE inhibitors used had suppressive effects on MLR despite differential PDE expression patterns among T cell populations [32] and distinct specificity of the inhibitors arguing for a general effect by this class of inhibitors. However, our studies using viable or fixed DC as stimulators clearly show that rolipram affects three distinct mechanisms that may be relevant in the suppression of MLR: a direct effect inhibiting IL-12 production by DC, another direct effect on responder T cell proliferation and interestingly, also an indirect effect that enhances the suppressive effect of T_reg_ cells. This particular mechanism is mediated by interfering with the activation of DC and their subsequent ability to prime alloreactive T cells in a cAMP dependent manner. We are unable to differentiate whether the elevated levels of cAMP in DC are truely achieved by the transfer via GJIC as suggested by our calcein assay and the decreased cAMP levels in suppressed DC in the presence of the GJIC inhibitor. On the other hand, the GJIC inhibitory peptide GAP27 can not inhibit all potential gap junction variants with equal efficiency [38]. Alternatively, T_reg_ cells may trigger other surface receptors, such as A_2A_ or A_2B_ receptors that in turn activate cAMP dependent signalling pathways [39]. However, using the A_2A_ receptor inhibitor SCH58261 in an MLR, we did not observe any enhancing or inhibitory effects on T cell proliferation (data not shown) arguing against a major role of this particular mechanism in the context of allogeneic T cell activation.

Conversely, our data allow the conclusion that T_reg_/DC communications require live cell interactions as fixed T_reg_ cells are unable to confer the suppressive phenotype (Fig. S3D). Importantly, under our experimental conditions where T_reg_ cells had been depleted from the T cell graft before transplantation (Fig. 6), we merely observed a moderate delay in the course of GvHD as illustrated by the survival plots when T_reg_ cells were transplanted alone. The same is true for rolipram alone in this situation, suggesting that the pronounced direct effect of rolipram on responder T cell proliferation might not be the most relevant mechanism of rolipram in suppression of GvHD *in vivo*. On the contrary, the fact that we observe nearly full protection in the group of T_reg_ transfer and rolipram suggests that a mechanism involving the cAMP dependent T_reg_/DC interaction is more important in the *in vivo* situation. However, we can not exclude off-target effects of rolipram *in vivo*, i.e. on PDE3 which is important for the induction of T_reg_ cells in the context of allograft rejection [31]. Here, we are unable to differentiate whether rolipram affects transplanted T_reg_ cells or mediates the induction of T_reg_ cells which is a limitation of our study.

Never the less, our findings are corroborated by the recent work of Klein and coworkers who demonstrate the cAMP in human T_reg_ cells is essential for T_reg_ mediated suppression in general and also in the context of GvHD using a humanized mouse model [40]. Finally, our results fit well with previous data from O’Shaughnessy et al. who demonstrate that elevated cAMP levels in CD4^+^ T cells are protective in GvHD [41]. We can now extend this notion by showing that this protection involves the enhanced suppressive action of T_reg_ cells indicating that one important pathway of T_reg_ mediated suppression is via a metabolic pathway involving cAMP. This pathway can be modulated by drugs like the phosphodiesterase inhibitor rolipram.

Taken together, our results provide the basis for an advanced understanding of T_reg_ mediated suppression of GvHD that may allow the establishment of combined approaches incorporating adoptive immunotherapy with targeted drug treatments to overcome the current limitations of allogeneic HSCT.

## Supporting Information

Figure S1 **T_reg_ cells induce a suppressive DC phenotype.** (A) Gating strategy to discriminate between BMDC and pre T_reg_ after coculture. (B) BALB/c BMDC were left untreated (DC, filled grey area) or cultured with C57BL/6 pre T_reg_ cells (DC_sup_, solid black line) in a 1∶1 ratio. Where indicated LPS (100 ng/ml) was added to the culture (solid black line). For optimal pre T_reg_ stimulation soluble anti-CD3-mAb (3 µg/ml) was added. After 4 h expression of CD80, B7-H1 and B7-DC was determined by flow cytometry.
(TIFF)

Figure S2 **Rolipram affects LPS induced IL-12 production by DC, but not the activation phenotype.** BALB/c splenic DC were left untreated (black bars) or stimulated with LPS (100 ng/ml, white bars) over night in presence of different concentrations of the PDE4 inhibitor rolipram (100 to 1000 nM). (A, B, C) Expression of the activation markers CD80, CD86 and CD40 was assessed by flow cytometry. (D) Release of IL-12 into the media was measured by a specific ELISA after 2 days of incubation. (E) Viability of the DC was determined after over night incubation by staining with propidium iodide (PI) and flowcytometric determination of the percentage of PI negative cells.
(TIFF)

Figure S3 **Fixed BMDC are sufficient stimulators in MLR.** (A) Scheme of the experimental setup. (B) C57BL/6 Thy1.2^+^ T cells were left untreated, cocultured with fixed BALB/c BMDC or viable BALB/c BMDC (1×10^4^ per well) in a 10∶1 ratio for 3 days. Proliferation was determined by ^3^H-thymidine incorporation. (C) BALB/c BMDC were stimulated with LPS (100 ng/ml) in absence or presence of C57BL/6 pre T_reg_ cells in a 1∶1 ratio for 4 h with soluble anti-CD3 (3 µg/ml). After fixation cells were cultured with viable C57BL/6 Thy1.2^+^ T cells in a 10∶1 T/DC ratio for 3 days. Proliferation was determined by ^3^H-thymidine incorporation. (D) BALB/c BMDC were left alone or cocultured with C57BL/6 Treg in a 1∶1 ratio for 4 h with soluble anti-CD3 (3 µg/ml). Without separation the cells were subsequently fixed and used as stimulators for C57BL/6 T cells (T/DC 10∶1) for 3 days. As a additional control C57BL/6 T cells were stimulated with viable BALB/c DC alone or together with fixed C57BL/6 Treg in the same ratios as stated above. Proliferation was determined by ^3^H-thymidine incorporation. All depicted results were assayed in six replicate wells and are representative for two independent experiments. (*) indicates significant differences by Mann-Whitney test. n.s. – no significant differences.
(TIFF)

Figure S4 **Rolipram enhances suppressive capacities of T_reg_ cells** ***in vivo*** **.** BALB/c mice were lethally irradiated (8,5 Gy) and received either TCD bone marrow (5×10^6^ cells) and Thy1.2^+^ CD25^−^ T cells (5×10^5^ cells) from C57BL/6 donors (n = 10, filled squares), Thy1.2^+^ T cells plus T_reg_ cells (1∶1 ratio, n = 10, open squares), Thy1.2^+^ T cells plus rolipram (n = 10, filled circles) or Thy1.2^+^ T cells plus T_reg_ cells and rolipram (n = 9, open circles). Rolipram (0,3 mg/kg) was injected *i.p.* on days 0 to 20 once a day. Results show the combined scoring data evaluated according to the clinical scoring system from 2 independent experiments.
(TIFF)
