# Supplementary figures and images for: Mechanisms of Cyclic Nucleotide Phosphodiesterases in Modulating T Cell Responses in Murine Graft-versus-Host Disease
Source: PLoS One. 2013 Mar 6;8(3):e58110. doi: 10.1371/journal.pone.0058110 (PMC3590136; doi:10.1371/journal.pone.0058110)

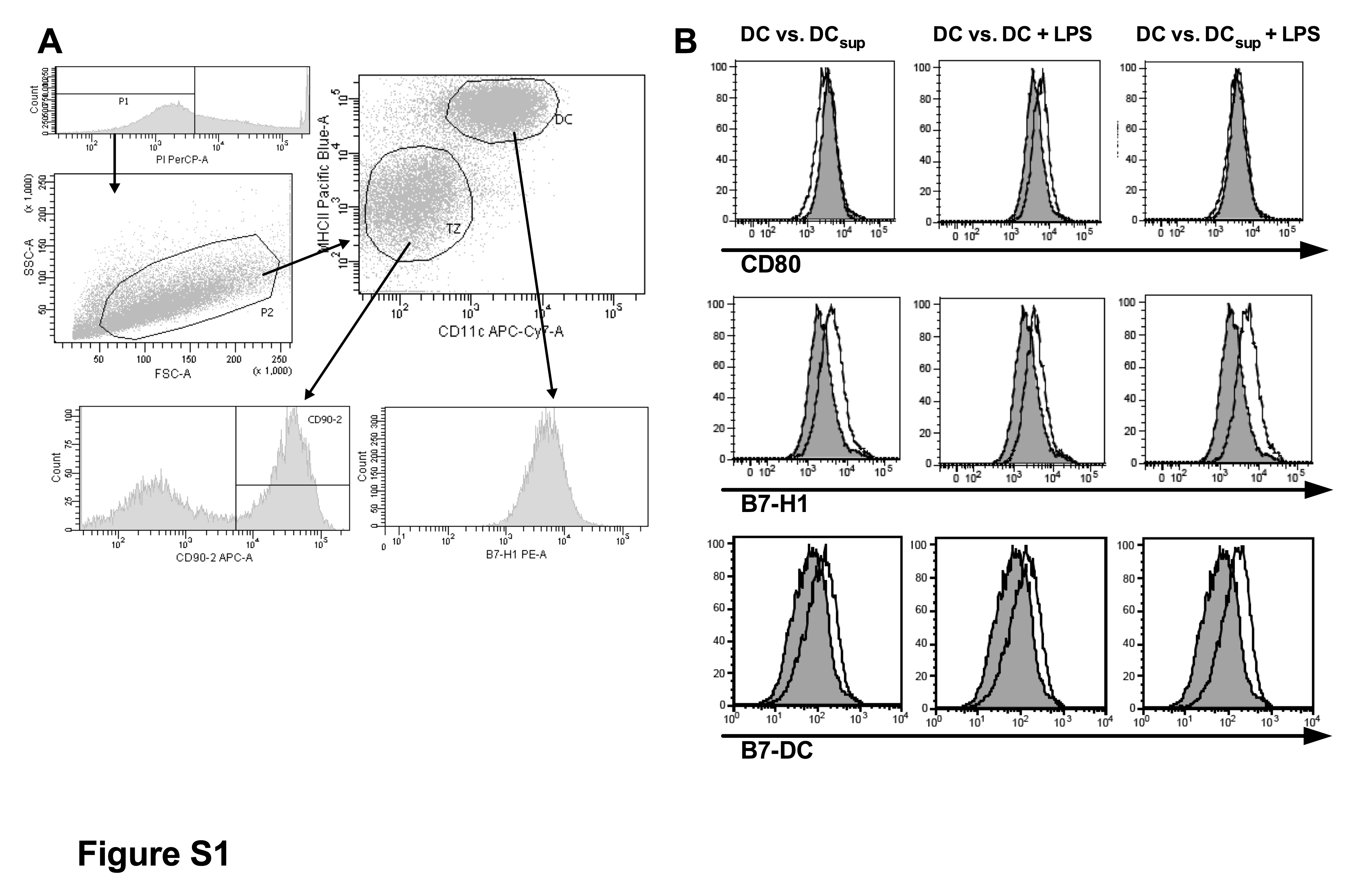

Supplement: Figure S1 — Treg cells induce a suppressive DC phenotype. (A) Gating strategy to discriminate between BMDC and pre Treg after coculture. (B) BALB/c BMDC were left untreated (DC, filled grey area) or cultured with C57BL/6 pre Treg cells (DCsup, solid black line) in a 1∶1 ratio. Where indicated LPS (100 ng/ml) was added to the culture (solid black line). For optimal pre Treg stimulation soluble anti-CD3-mAb (3 µg/ml) was added. After 4 h expression of CD80, B7-H1 and B7-DC was determined by flow cytometry. (TIFF) [file pone.0058110.s001.tiff]

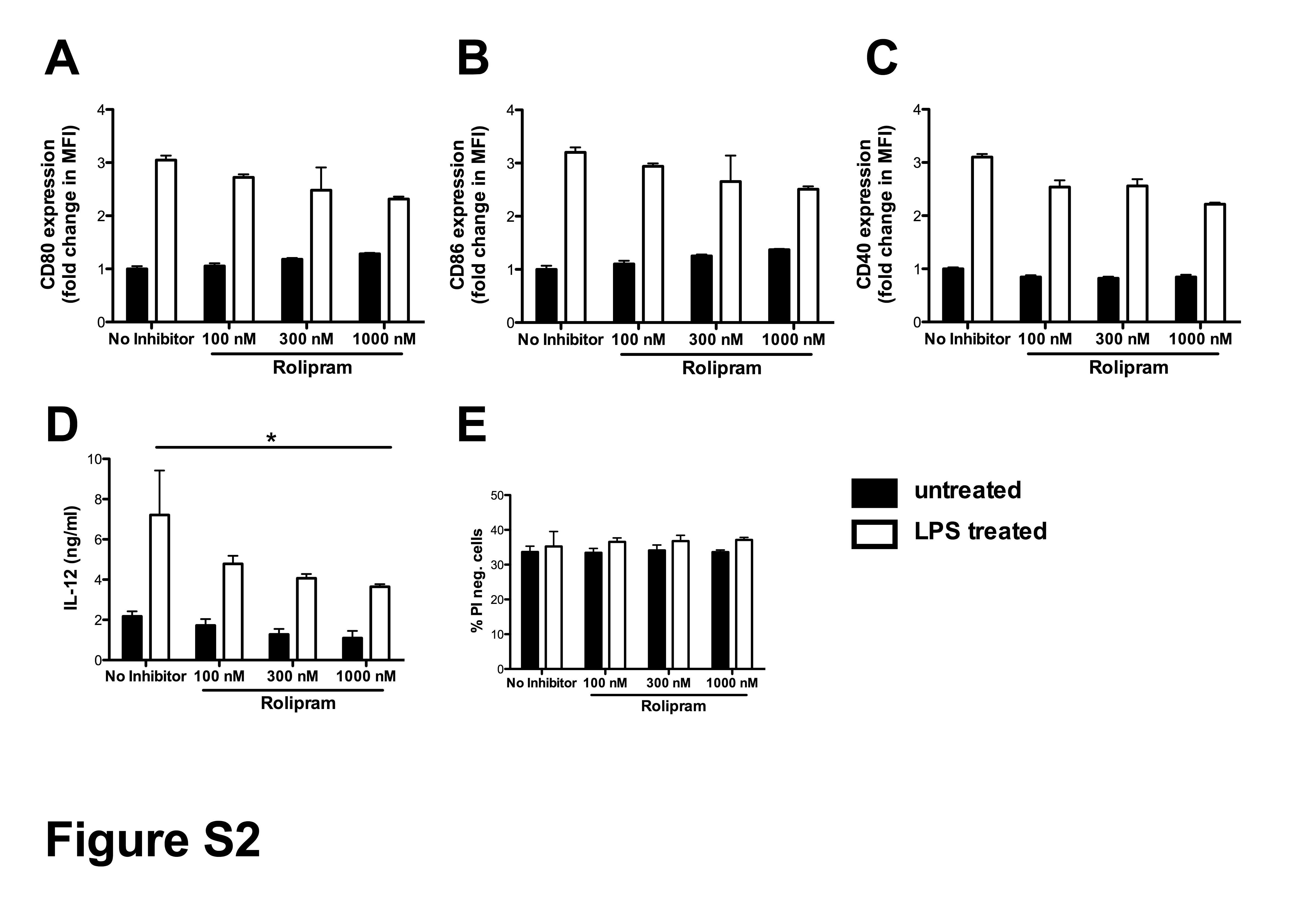

Supplement: Figure S2 — Rolipram affects LPS induced IL-12 production by DC, but not the activation phenotype. BALB/c splenic DC were left untreated (black bars) or stimulated with LPS (100 ng/ml, white bars) over night in presence of different concentrations of the PDE4 inhibitor rolipram (100 to 1000 nM). (A, B, C) Expression of the activation markers CD80, CD86 and CD40 was assessed by flow cytometry. (D) Release of IL-12 into the media was measured by a specific ELISA after 2 days of incubation. (E) Viability of the DC was determined after over night incubation by staining with propidium iodide (PI) and flowcytometric determination of the percentage of PI negative cells. (TIFF) [file pone.0058110.s002.tiff]

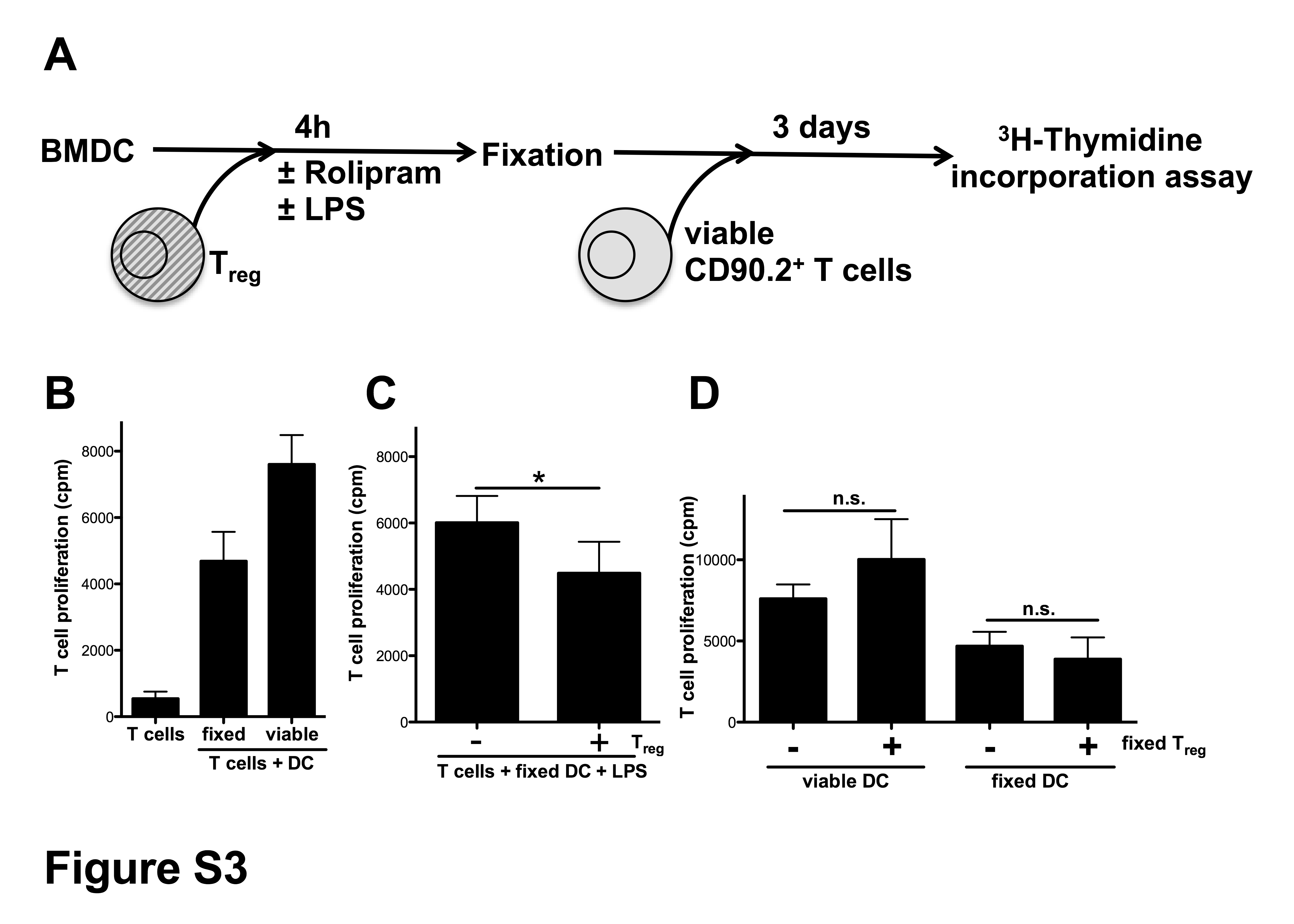

Supplement: Figure S3 — Fixed BMDC are sufficient stimulators in MLR. (A) Scheme of the experimental setup. (B) C57BL/6 Thy1.2+ T cells were left untreated, cocultured with fixed BALB/c BMDC or viable BALB/c BMDC (1×10^4^ per well) in a 10∶1 ratio for 3 days. Proliferation was determined by 3H-thymidine incorporation. (C) BALB/c BMDC were stimulated with LPS (100 ng/ml) in absence or presence of C57BL/6 pre Treg cells in a 1∶1 ratio for 4 h with soluble anti-CD3 (3 µg/ml). After fixation cells were cultured with viable C57BL/6 Thy1.2+ T cells in a 10∶1 T/DC ratio for 3 days. Proliferation was determined by 3H-thymidine incorporation. (D) BALB/c BMDC were left alone or cocultured with C57BL/6 Treg in a 1∶1 ratio for 4 h with soluble anti-CD3 (3 µg/ml). Without separation the cells were subsequently fixed and used as stimulators for C57BL/6 T cells (T/DC 10∶1) for 3 days. As a additional control C57BL/6 T cells were stimulated with viable BALB/c DC alone or together with fixed C57BL/6 Treg in the same ratios as stated above. Proliferation was determined by 3H-thymidine incorporation. All depicted results were assayed in six replicate wells and are representative for two independent experiments. (*) indicates significant differences by Mann-Whitney test. n.s. – no significant differences. (TIFF) [file pone.0058110.s003.tiff]

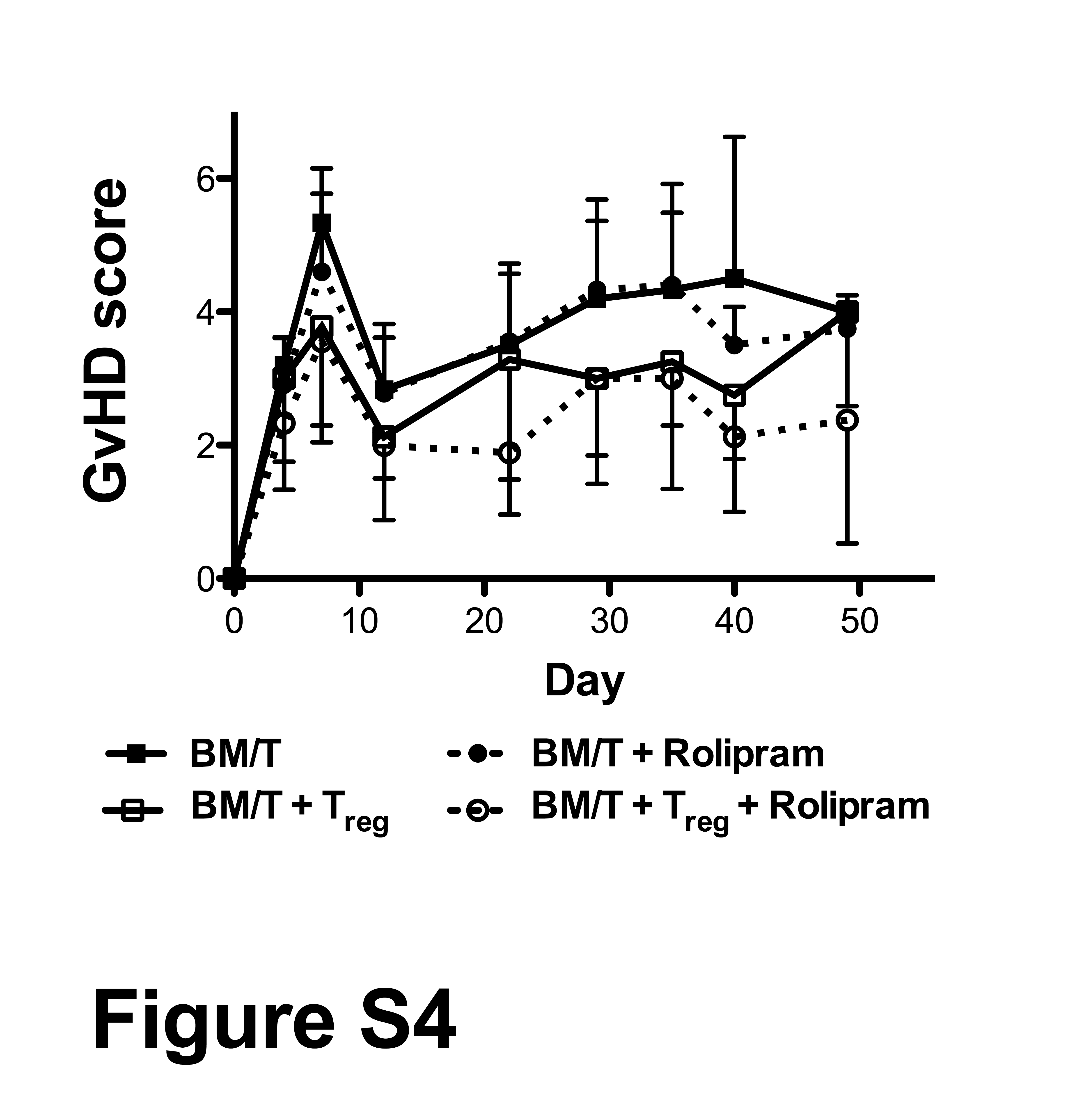

Supplement: Figure S4 — Rolipram enhances suppressive capacities of Treg cells in vivo . BALB/c mice were lethally irradiated (8,5 Gy) and received either TCD bone marrow (5×10^6^ cells) and Thy1.2+ CD25− T cells (5×10^5^ cells) from C57BL/6 donors (n = 10, filled squares), Thy1.2+ T cells plus Treg cells (1∶1 ratio, n = 10, open squares), Thy1.2+ T cells plus rolipram (n = 10, filled circles) or Thy1.2+ T cells plus Treg cells and rolipram (n = 9, open circles). Rolipram (0,3 mg/kg) was injected i.p. on days 0 to 20 once a day. Results show the combined scoring data evaluated according to the clinical scoring system from 2 independent experiments. (TIFF) [file pone.0058110.s004.tiff]
